# Life-Course Noncardiac Comorbidity Burden and Mortality in Lower-Complexity Congenital Heart Disease

**DOI:** 10.1016/j.jacadv.2026.103278

**Published:** 2026-09-24

**Authors:** Jie-Xin Zhang, Meng-Yi Liu, Pei-Er Chen, Jia-Xing Zhang, Guoli Sun, Shen-Shen Huang, Kai Zhang, Cai-Wen Ou, Shu-Shui Wang, Zhi-Cheng Jing

**Affiliations:** a Department of Cardiology, Peking Union Medical College Hospital, Chinese Academy of Medical Sciences and Peking Union Medical College, Beijing, China; b Department of Cardiovascular Medicine, Guangdong Cardiovascular Institute, Guangdong Provincial People's Hospital, Guangdong Academy of Medical Sciences, State Key Laboratory of Multi-organ Injury Prevention and Treatment, Southern Medical University, Guangzhou, China; c The Tenth Affiliated Hospital (Dongguan People's Hospital), Southern Medical University, Dongguan, China; d Guangdong Provincial Key Laboratory of Cardiac Function and Microcirculation, Guangzhou, China; e Department of Paediatric Cardiology, Guangdong Provincial People’s Hospital, Guangdong Academy of Medical Sciences, Southern Medical University, Guangzhou, China

**Keywords:** congenital heart disease, lower-complexity, noncardiac comorbidity, lifetime risk, long-term mortality

## Abstract

**Background:**

Lower-complexity congenital heart disease (CHD) is generally perceived as a low-risk condition. However, its long-term systemic comorbidity burden and impact on survival remain poorly defined, despite increasing recognition of lifelong care needs.

**Objectives:**

The authors aimed to determine whether lower-complexity CHD is a life-course condition characterized by high noncardiac comorbidity burden and mortality among adults.

**Methods:**

This population-based prospective cohort study identified 3,316 adults with lower-complexity CHD and 492,232 individuals without CHD from the UK Biobank. The primary outcome was the first occurrence of 9 system-specific noncardiac comorbidities, with relative rates estimated by multivariable Cox regression models and lifetime risk from age 40 to 90 assessed using life-table methods. The secondary outcome was all-cause mortality among individuals with lower-complexity CHD, assessed according to baseline and incident comorbidity status using time-varying Cox regression models. The median follow-up was approximately 13 years.

**Results:**

Compared to individuals without CHD, adults with lower-complexity CHD had significantly elevated relative rates of noncardiac comorbidities across 9 organ systems, particularly for blood, endocrine, and pulmonary diseases. Comorbidity incidence diverged from midlife and accelerated with age. Absolute lifetime risks ranged from 17.1% (skin disease) to 45.8% (digestive disease), with the largest excess risks observed in endocrine (18.6%), digestive (15.7%), and pulmonary (12.5%) systems. Among individuals with CHD, incident noncardiac comorbidities were independently associated with increased subsequent all-cause mortality, with pulmonary disease conferring the strongest relative rate.

**Conclusions:**

Lower-complexity CHD is associated with higher lifetime noncardiac comorbidity burden and increased subsequent mortality. These findings support a shift toward integrated, life-course clinical management.

Congenital heart disease (CHD), affecting around 0.8% of live births, is the most common congenital anomaly.^1^ Advances in pediatric care now enable over 90% of patients to reach adulthood,^2^^,^^3^ shifting CHD-related mortality from childhood to adulthood.^4^ Lower-complexity CHD is often regarded as a low-risk condition, with generally favorable long-term outcomes and less intensive follow-up requirements reported in prior studies.^5^ However, existing evidence has predominantly focused on cardiac outcomes, while the broader systemic health trajectory of these individuals remains insufficiently characterized.

Emerging evidence suggests that adults with CHD may experience an increased burden of noncardiac comorbidities, reflecting a more complex and systemic disease process.^6^ In contrast to patients with complex lesions who often die from underlying CHD, those with lower-complexity CHD tend to live longer and die from non-CHD conditions.^7^, ^8^, ^9^ Recent guideline updates have emphasized the need for lifelong, multidisciplinary care in CHD.^10^ Nevertheless, several critical gaps remain. First, the life-course trajectory and absolute burden of noncardiac comorbidities in lower-complexity CHD have not been well quantified. Second, the extent to which incident noncardiac comorbidities contribute to subsequent mortality in this population is poorly understood.

As the number of CHD patients over the age of 65 years continues to rise,^11^ the UK Biobank (UKB), a prospective cohort of over 500,000 individuals with long-term follow-up and high late-life survival,^12^ provides an opportunity to assess how noncardiac comorbidities develop and affect long-term outcomes in adults with lower-complexity CHD. In this context, we used UKB to examine the life-course burden of noncardiac comorbidities and their implications for hazards of mortality.

## Methods

### Study design and data source

We conducted a population-based prospective cohort study using the UKB, which recruited over 500,000 participants aged 37 to 73 years with linked health records. All details about the cohort have been described previously.^13^^,^^14^ The UKB received ethical approval from the North West Multicenter Research Ethics Committee, and all participants provided written informed consent. Studies using the deidentified UKB data are exempt from additional institutional review.

#### Study population

Data from 502,140 participants were available for our study. Informed by previously validated UKB algorithms reported by Saha et al,^12^ a stepwise classification algorithm (Figure 1) was applied to identify 3,359 individuals with CHD and 492,232 without CHD for comparison. We identified the population based on International Classification of Diseases (ICD)-9, ICD-10, and Office of Population, Censuses and Surveys: Classification of Interventions and Procedures (OPCS)-4 codes from hospital records as well as self-reported data. All code lists are detailed in Supplemental Tables 1 to 4.

**Figure 1:**
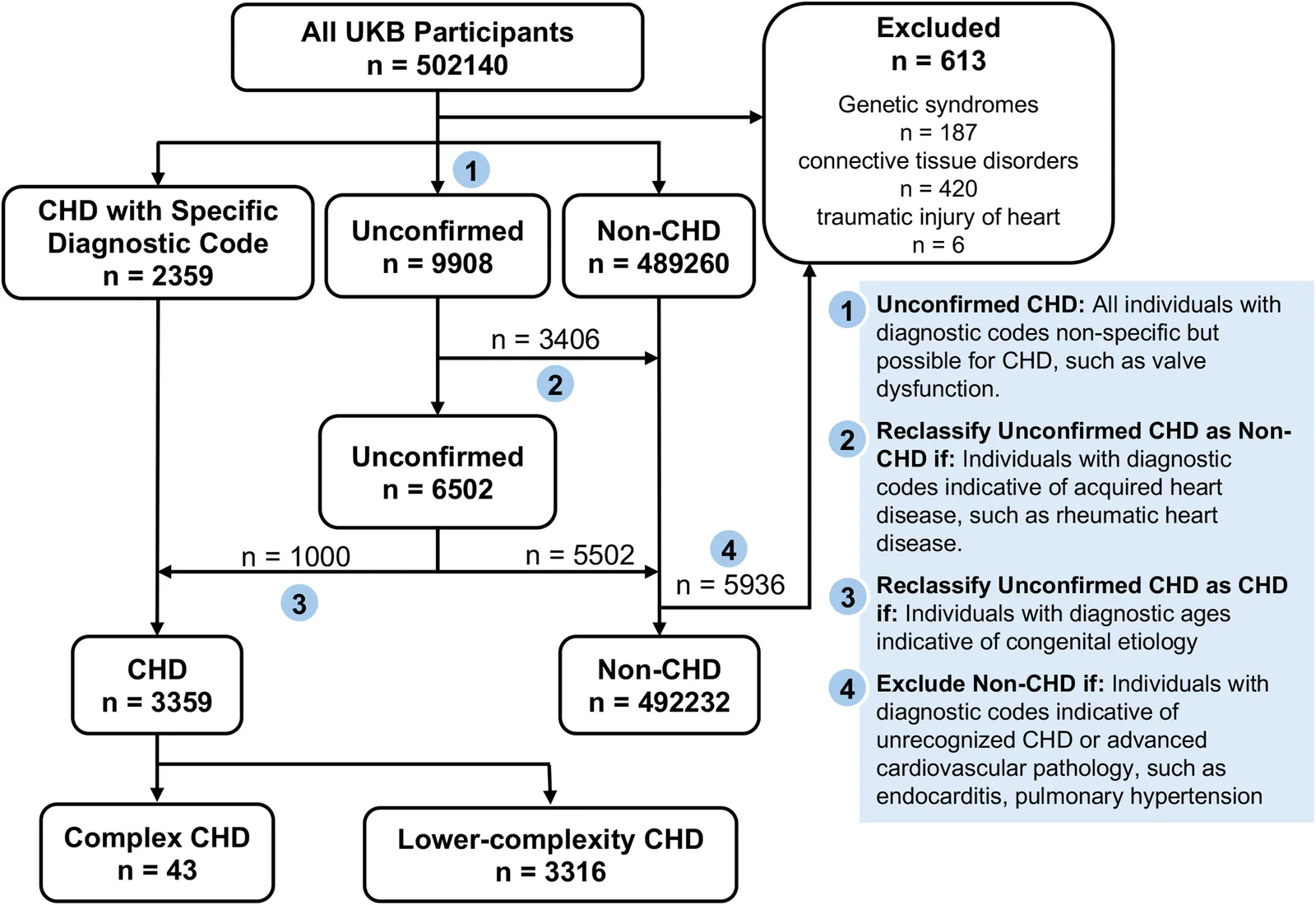
Classification Algorithm Flowchart for Defining Individuals With and Without Congenital Heart Disease in UK Biobank Diagnostic codes (ICD9, ICD10, OPCS-4, and self-reported data) for defining each group are given in Supplemental Tables 1 to 4. CHD = congenital heart disease; UKB = UK Biobank.

Lesion stratification was based primarily on anatomic complexity, supplemented by hemodynamic burden. Complex CHD (n = 43) represents great complexity CHD classified by the most recent American College of Cardiology (ACC)/American Heart Association (AHA) guideline for adults with CHD,^10^ along with lesions with unspecified cyanosis. Lower-complexity CHD (n = 3,316, 100%) is comprised of simple CHD, moderate-complexity CHD, and other lower-complexity CHD. Simple CHD accounted for 1,055 (31.8%) individuals, including left-to-right shunt (n = 1,036, 31.2%) and pulmonic stenosis (n = 19, 0.6%). Left-to-right shunt included atrial septal defect, ventricular septal defect, and patent ductus arteriosus. Moderate-complexity CHD (n = 1,821, 54.9%) comprised congenital aortic valve disease, congenital mitral valve disease, coarctation of the aorta, Ebstein anomaly, peripheral pulmonary artery stenosis, anomalous pulmonary venous connection, atrioventricular septal defect, and tetralogy of Fallot, and they were further categorized as left-sided lesions (n = 1,421, 42.9%), right-sided lesions (n = 240, 7.2%), and other moderate-complexity lesions which included shunt lesions (n = 160, 4.8%). Congenital abnormalities not explicitly listed in the 2025 ACC/AHA guideline classification were assigned according to their anatomic involvement. For example, congenital pulmonary valve insufficiency was classified as a right-sided lesion. Lesions that could not be reliably assigned to a specific anatomical category, including conduction defect, dextrocardia, and levocardia (n = 65, 2.0%), along with unspecified CHD (n = 375, 11.3%) were classified as other lower-complexity CHD (n = 440, 13.3%). For participants with multiple lesions, the most complex anatomic diagnosis determines the anatomic complexity according to the 2025 ACC/AHA guideline. Subgroup definitions are provided in Supplemental Table 5.

Although CHD is present from birth, the UKB cohort represents a delayed-entry population. Accordingly, time-to-event analyses were left-truncated at age at enrollment, and participants with pre-existing diagnoses of the outcome were excluded to estimate risk trajectories from midlife onward. Due to system-specific case definitions and data completeness, the number of participants and follow-up duration varied by system (Supplemental Table 6). Individuals were included in each system-specific analysis only if they had nonmissing information relevant to that system.

## Outcomes

The primary outcome was the first occurrence of system-specific noncardiac comorbidities across 9 organ systems (pulmonary, neurological, mental, genitourinary, digestive, musculoskeletal, endocrine, skin, and blood-related). Each organ system was treated as an independent and parallel outcome domain due to their distinct pathophysiologic profiles, and no composite endpoint was constructed. Diagnoses were ascertained using ICD-10 codes from hospital admissions, primary care, death register, and self-reports in UKB (Supplemental Table 7).

The secondary outcome was all-cause mortality among individuals with lower-complexity CHD. Mortality data were obtained from the death register.

### Relative rate analysis for noncardiac comorbidities

For the primary outcome, multivariable Cox proportional hazards models were used to estimate HRs and 95% CIs, with lower-complexity CHD status as the primary exposure. Models were adjusted for age at recruitment, sex, ethnicity (Caucasian vs non-Caucasian), Townsend Deprivation Index,^15^ body mass index, smoking status (never, former, current), alcohol consumption (daily, weekly, seldom), and physical activity (low vs high, with “high” defined as at least 3 days per week of vigorous-intensity physical activity or 5 days per week of moderate-intensity physical activity).^12^^,^^16^ Kaplan-Meier curves were used for descriptive comparison, assessed using the log-rank test.

### Lifetime risk analysis for noncardiac comorbidities

For the primary outcome, censoring occurred at the earliest of outcome diagnosis, death, loss to follow-up, or the end of regional data availability. Absolute lifetime risk, estimated by the life-table methods, was defined as the absolute probability of developing a system-specific comorbidity from age 40 to 90, with death treated as a competing event. Age at entry and age at outcome or censoring were used to construct 5-year age–stratified intervals for cumulative risk estimation, with age-specific incidence rates derived using a person-years approach. Uncertainty was quantified using bootstrap sampling to derive 95% CIs and absolute risk differences between individuals with and without lower-complexity CHD.

### All-cause mortality analysis

For the secondary outcome, follow-up time was defined from baseline to death, loss to follow-up, or the end of regional data availability. Cumulative survival probabilities and 95% CIs were estimated using Kaplan-Meier curves, stratified by the presence of noncardiac comorbidities. In primary models, comorbidities were treated as baseline exposures. Multivariable Cox proportional hazards models were fitted to quantify the association between noncardiac comorbidities and all-cause mortality among individuals with lower-complexity CHD. In secondary analyses, we extended the Cox proportional hazard model with time-varying exposure,^17^ in which comorbidity status was treated as a time-dependent covariate and updated over follow-up, to estimate the association between incident comorbidity and subsequent mortality. This time-varying approach minimizes potential immortal time bias by ensuring temporal alignment between exposure status and comorbidity onset. The same covariates were used as above.

### Sensitivity analyses

To assess the potential impact of lesion heterogeneity and exposure misclassification, multivariable Cox proportional hazards models were repeated separately for participants with simple CHD and moderate-complexity CHD to evaluate the HRs of developing system-specific noncardiac comorbidities using the same covariate adjustments as above. To evaluate the potential influence of intervention, we identified participants with evidence of intervention records by OPCS-4 codes from hospital records as well as self-reported data (Supplemental Table 10). Multivariable Cox proportional hazards models were performed for both participants with and without identifiable intervention records. Additionally, E-value analyses were performed to assess the degree of residual confounding that would be needed to reverse the associations. Competing-risk analyses using Fine–Gray subdistribution hazard models were performed as sensitivity analyses for noncardiac comorbidity outcomes, with death treated as a competing event.^18^

### Statistical analysis

Baseline characteristics were summarized by CHD status and CHD subgroups. Categorical variables were presented as frequencies and percentages and compared using chi-squared test. Continuous variables, assumed to be non-normally distributed, were reported as medians with IQRs and compared using the Wilcoxon rank sum test.

To visualize system-specific associations, estimates from life-table analysis and Cox models of each system were combined into forest plots. All statistical analyses were conducted in R version 4.4.1. *P* value <0.05 was considered statistically significant.

## Results

### Characteristics of study population

A total of 495,548 participants were included, comprising 492,232 individuals without CHD and 3,316 with lower-complexity CHD. Among the latter, 1,055 had simple CHD, 1,821 had moderate-complexity CHD, and 440 had other lower-complexity CHD.

Baseline demographic characteristics are presented in Table 1. A lower proportion of lower-complexity CHD individuals were female (43.4% vs 54.6%, *P* < 0.001). Most participants were of Caucasian ethnicity (>94%), with slightly higher proportions in the lower-complexity CHD group. Lower-complexity CHD individuals were more socioeconomically deprived (*P* < 0.001), with a median Townsend index of −1.87 (IQR: −3.50 to 1.08), compared to −2.14 (IQR: −3.65 to 0.54) in non-CHD participants. Individuals with lower-complexity CHD had a higher body mass index (27.27, IQR: 24.65-30.78) than non-CHD participants (26.73, IQR: 24.13-29.88) (*P* < 0.001). Behavioral characteristics also differed (Table 1). Lower-complexity CHD individuals had a higher proportion of current or former smokers (48.2% vs 45.1%, *P* = 0.002) but seldom drinkers (22.4% vs 19.6%, *P* < 0.001). Physical activity levels were comparable across groups (*P* = 0.267).

**Table 1:** Characteristics of the Study Population at Enrollment by CHD Status and Subgroups

	Non-CHD (n = 492,232)	Lower-Complexity CHD (n = 3,316)	Simple CHD (n = 1,055)	Moderate-Complexity CHD (n = 1,821)	Other Lower-Complexity CHD (n = 440)	*P* Value
Demographics						
Age at enrollment, y, median [IQR]	58 [50-63]	58 [50-63]	58 [50-64]	58 [50-63]	57 [50-63]	0.862
Female, n (%)	268,761 (54.6)	1,438 (43.4)	554 (52.5)	661 (36.3)	223 (50.7)	<0.001
Caucasian ethnicity, n (%)	463,063 (94.6)	3,147 (95.5)	1,001 (95.5)	1,726 (95.6)	420 (95.5)	0.019
Townsend Deprivation Index, median [IQR]	−2.14 [−3.65 to 0.54]	−1.87 [−3.50 to 1.08]	−1.98 [−3.56 to 0.71]	−1.84 [−3.47 to 1.11]	−1.69 [−3.48 to 1.78]	<0.001
Body mass index, kg/m^2^, median [IQR]	26.73 [24.13-29.88]	27.27 [24.65-30.78]	26.89 [24.26-30.17]	27.59 [24.83-31.17]	27.00 [24.31-30.45]	<0.001
Behavioral risk factors, n (%)						
Smoking						0.002
Never	268,614 (54.9)	1,706 (51.8)	574 (54.7)	896 (49.6)	236 (53.8)	
Former	169,032 (34.5)	1,220 (37.0)	383 (36.5)	683 (37.8)	154 (35.1)	
Current	51,713 (10.6)	370 (11.2)	92 (8.8)	229 (12.7)	49 (11.2)	
Alcohol consumption						<0.001
Daily	213,133 (43.4)	1,358 (41.1)	422 (40.2)	770 (42.4)	166 (37.7)	
Weekly	181,553 (37.0)	1,208 (36.5)	385 (36.6)	642 (35.4)	181 (41.1)	
Seldom	96,083 (19.6)	740 (22.4)	244 (23.2)	403 (22.2)	93 (21.1)	
Physical activity						0.267
High	242,766 (52.6)	1,574 (51.6)	530 (54.2)	860 (51.4)	184 (45.8)	
Low	218,959 (47.4)	1,479 (48.4)	447 (45.8)	814 (48.6)	218 (54.2)	

Comparisons were considered statistically significant when *P* < 0.05.

Number of participants do not sum to group totals due to missing values.

CHD = congenital heart disease.

## HRs and cumulative incidence of noncardiac comorbidities

Multivariable Cox proportional hazards models showed significantly elevated relative rates of comorbidities across all 9 noncardiac systems. The strongest associations were observed in blood disease (HR: 2.43; 95% CI: 2.24-2.63), endocrine disease (HR: 2.30; 95% CI: 2.16-2.44), and pulmonary disease (HR: 2.30; 95% CI: 2.14-2.46). Skin and subcutaneous disease had the smallest HR (HR: 1.26; 95% CI: 1.15-1.37) (Table 2). Within each noncardiac system, the 6 most frequent diagnoses with their event counts and proportions among participants with lower-complexity CHD were detailed in Supplemental Table 7. Representative diagnoses included anemias, purpura, agranulocytosis, and coagulation defects within blood disease; disorders of lipoprotein metabolism, obesity, type 2 diabetes mellitus and fluid, electrolyte, and acid-base disorders within endocrine disease; acute lower respiratory infection, pleural effusion, pneumonia, chronic obstructive pulmonary disease, and asthma within pulmonary disease. In the remaining systems, frequent diagnoses included sleep disorders and transient cerebral ischemic attacks within neurologic disease; tobacco-related mental and behavioral disorders and depressive episode within mental and behavioral disease; diverticular disease of intestine and gastroesophageal reflux disease within digestive disease; acute and chronic renal failure within genitourinary disease; cellulitis and dermatitis within skin and subcutaneous disease; arthrosis and soft tissue disorders within musculoskeletal disease. Due to variation in outcome definitions and data completeness, median follow-up time ranged from 12.76 to 13.54 years across 9 systems (Supplemental Table 6). Kaplan-Meier analyses demonstrated significantly higher cumulative incidence of noncardiac comorbidities across 9 systems among individuals with lower-complexity CHD compared to those without CHD (Supplemental Figure 1). The E-values suggested that relatively strong unmeasured confounding would be required to reverse the associations shown above (Supplemental Table 8). Fine–Gray subdistribution hazard models, to account for the competing risk of death, demonstrated broadly consistent associations compared with the primary Cox proportional hazards analyses (Supplemental Table 9).

**Table 2:** Adjusted HRs and 95% CIs of First-Time Noncardiac Comorbidities by System in Individuals With Lower-Complexity CHD, Simple CHD, or Moderate-Complexity CHD

	Comparison	No. of Events/No. of Participants	HR (95% CI)	*P* Value
Neurologic disease	Non-CHD (control)	59,165/418,803	1.00	Ref
	Lower-complexity CHD	641/2,561	1.80 (1.66-1.96)	<0.001
	Simple CHD	205/779	2.00 (1.73-2.31)	<0.001
	Moderate-complexity CHD	356/1,439	1.74 (1.55-1.94)	<0.001
Pulmonary disease	Non-CHD (control)	**69,507/384,640**	**1.00**	**Ref**
	Lower-complexity CHD	**904/2,363**	**2.30 (2.14**-**2.46)**	**<0.001**
	Simple CHD	257/757	2.15 (1.89-2.45)	<0.001
	Moderate-complexity CHD	565/1,302	2.57 (2.35-2.81)	<0.001
Endocrine disease	Non-CHD (control)	**96,804/367,632**	**1.00**	**Ref**
	Lower-complexity CHD	**1,117/2,167**	**2.30 (2.16**-**2.44)**	**<0.001**
	Simple CHD	337/698	2.15 (1.92-2.41)	<0.001
	Moderate-complexity CHD	652/1,165	2.54 (2.34, 2.76)	<0.001
Mental and behavioral disease	Non-CHD (control)	**59,088/408,557**	**1.00**	**Ref**
	Lower-complexity CHD	**617/2,675**	**1.71 (1.57**-**1.86)**	**<0.001**
	Simple CHD	180/853	1.64 (1.41-1.92)	<0.001
	Moderate-complexity CHD	355/1,475	1.77 (1.58-1.98)	<0.001
Digestive disease	Non-CHD (control)	**124,451/310,479**	**1.00**	**Ref**
	Lower-complexity CHD	**1,024/1,904**	**1.47 (1.37**-**1.56)**	**<0.001**
	Simple CHD	343/641	1.46 (1.30-1.63)	<0.001
	Moderate-complexity CHD	561/1,028	1.50 (1.37-1.63)	<0.001
Genitourinary disease	Non-CHD (control)	**85,790/330,414**	**1.00**	**Ref**
	Lower-complexity CHD	**895/2,198**	**1.66 (1.55**-**1.78)**	**<0.001**
	Simple CHD	276/682	1.66 (1.46-1.88)	<0.001
	Moderate-complexity CHD	509/1,226	1.69 (1.54-1.86)	<0.001
Blood disease	Non-CHD (control)	**47,973/466,187**	**1.00**	**Ref**
	Lower-complexity CHD	**720/3,014**	**2.43 (2.24**-**2.63)**	**<0.001**
	Simple CHD	220/945	2.40 (2.09-2.77)	<0.001
	Moderate-complexity CHD	432/1,667	2.61 (2.35-2.90)	<0.001
Skin and subcutaneous disease	Non-CHD (control)	**70,853/363,962**	**1.00**	**Ref**
	Lower-complexity CHD	**594/2,422**	**1.26 (1.15**-**1.37)**	**<0.001**
	Simple CHD	181/761	1.23 (1.05-1.43)	0.009
	Moderate-complexity CHD	338/1,344	1.28 (1.14-1.44)	<0.001
Musculoskeletal disease	Non-CHD (control)	**98,108/281,682**	**1.00**	**Ref**
	Lower-complexity CHD	**835/1,794**	**1.45 (1.35**-**1.55)**	**<0.001**
	Simple CHD	275/582	1.42 (1.26-1.61)	<0.001
	Moderate-complexity CHD	448/966	1.47 (1.33-1.62)	<0.001

Models were adjusted for age, sex, ethnicity, Townsend Deprivation Index, body mass index, smoking status, alcohol consumption, and physical activity. Comparisons were considered statistically significant when *P* < 0.05. **Bold** indicates the results of the overall analysis for lower complexity CHD.

Abbreviation as in Table 1.

In subgroup-stratified analyses, both participants with simple and moderate complexity CHD had significantly elevated relative rates of noncardiac comorbidities compared to non-CHD participants (Table 2). A total of 1,011 participants had identifiable intervention records, and their distribution across lower-complexity CHD subgroups has been summarized in Supplemental Table 10. In sensitivity analyses stratified by identifiable intervention records, the increased relative rates of noncardiac comorbidities remained significant across all systems regardless of intervention status, except for skin and subcutaneous diseases among participants with intervention records (Supplemental Table 11).

### Absolute lifetime risks of noncardiac comorbidities

Age-specific incidence rates of noncardiac comorbidities by system were higher and showed an earlier and steeper rise among lower-complexity CHD patients compared to non-CHD participants from age 40 to 85. In most systems, incidence rates diverged from midlife and increased progressively with advancing age, indicating an accelerated accumulation of systemic comorbidity burden in the CHD group, particularly after age 70 (Supplemental Figure 2).

Among lower-complexity CHD patients, the lifetime risks of noncardiac comorbidities ranged from 17.1% (95% CI: 15.2%-19.2%) for skin and subcutaneous disease to 45.8% (95% CI: 41.1%-51.5%) for digestive disease, and absolute risks from age 40 to 90 were significantly higher than those in non-CHD individuals across 8 systems, except skin and subcutaneous disease. The largest risk differences were observed for endocrine diseases (18.6%; 95% CI: 12.2%-24.3%), digestive diseases (15.7%; 95% CI: 11.1%-21.5%), and pulmonary diseases (12.5%; 95% CI: 7.8%-16.6%) (Figure 2).

**Figure 2:**
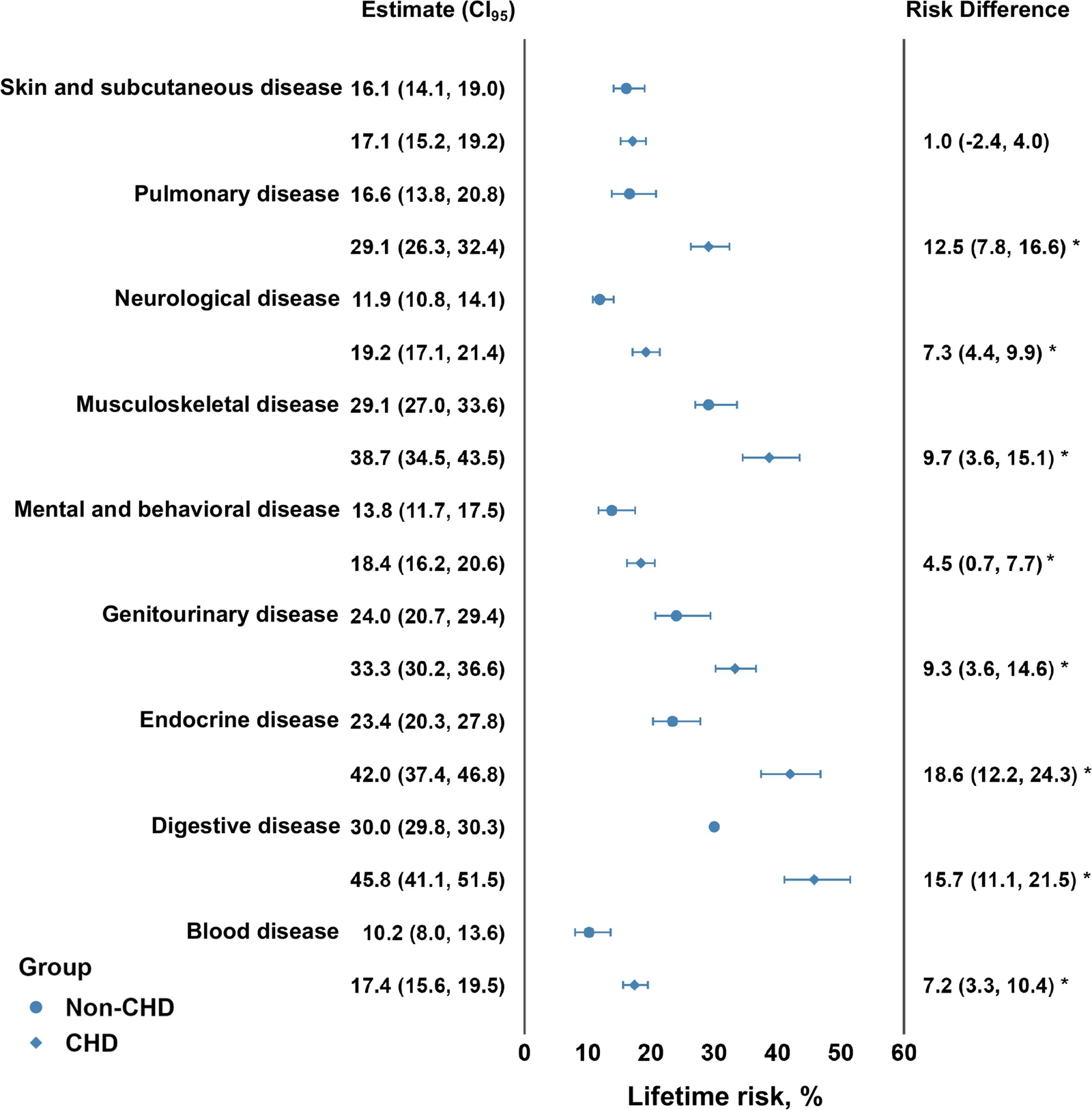
Forest Plot Showing the Absolute Lifetime Risk Estimates and 95% CIs of First-Time Noncardiac Comorbidities by System in Individuals With or Without Lower-Complexity Congenital Heart Disease From Age 40 to 90 Years Risk difference depicts excess risk among CHD patients compared with non-CHD individuals, and an asterisk (∗) after the 95% CI depicts the risk difference is statistically significant. Abbreviation as in Figure 1.

### Long-term survival of lower-complexity CHD patients

During follow-up, mortality event counts among individuals with lower-complexity CHD ranged from 317 to 571 across system-specific survival analyses (Supplemental Table 12). Kaplan-Meier analysis showed lower cumulative survival in those with noncardiac comorbidities than in those without, except for musculoskeletal disease (Supplemental Figure 3).

To quantify this effect, multivariable Cox models, treating comorbidities as baseline exposures and adjusting for the same covariates as above, showed the presence of noncardiac comorbidities was independently associated with increased mortality across 8 systems, except for musculoskeletal disease (HR: 0.94; 95% CI: 0.74-1.20). The strongest association was observed in pulmonary disease (HR: 3.38; 95% CI: 2.65-4.31) (Figure 3). When modeled as time-varying exposures, the onset of comorbidities during follow-up was associated with increased subsequent mortality across all 9 systems, with the strongest association also observed for pulmonary disease (HR: 7.93; 95% CI: 6.15-10.23) (Supplemental Figure 4).

**Figure 3:**
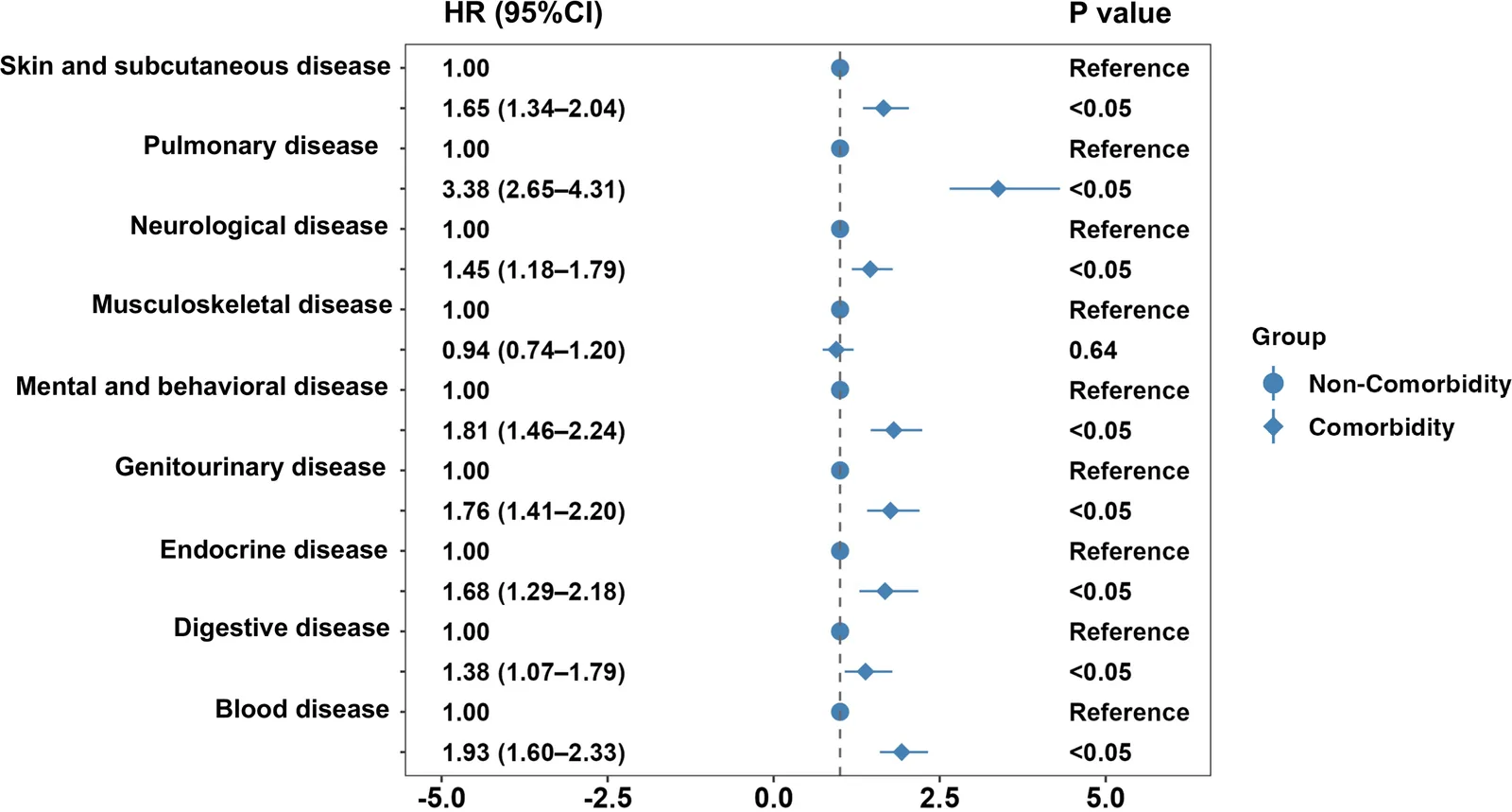
Forest Plot Showing the Adjusted HRs and 95% CIs of All-Cause Mortality Among Lower-Complexity Congenital Heart Disease Patients With or Without Noncardiac Comorbidities Across 9 Systems Models were adjusted for age, sex, ethnicity, Townsend Deprivation Index, body mass index, smoking status, alcohol consumption, and physical activity. Comparisons were considered statistically significant when *P* < 0.05.

## Discussion

In this population-based study, we demonstrate that participants with lower-complexity CHD, across both simple and moderate complexity lesions, had higher relative rates of system-specific noncardiac comorbidities compared to non-CHD participants. Comparable associations were also observed across subgroups stratified by identifiable intervention status, supporting the robustness of the primary findings. In line with a prior nationwide study demonstrating increased lifetime risks of comorbidities in simple CHD,^19^ our study moves beyond these observations by characterizing the coordinated, life-course accumulation of multisystem noncardiac comorbidities from midlife onward and linking it to higher subsequent mortality. The highest relative rates were observed for blood, endocrine, and pulmonary diseases, suggesting heightened multisystem susceptibility, whereas the greatest absolute lifetime risk differences occurred in the endocrine, digestive, and pulmonary systems, reflecting a substantial cumulative population-level burden driven by age-related accumulation. These findings align with the recent guideline,^10^^,^^20^ emphasizing the importance of lifelong, multidisciplinary care beyond lesion-specific cardiac outcomes in CHD and provide population-level evidence of a coordinated, multi-organ vulnerability in this population, highlighting midlife as a critical window for surveillance (Central Illustration).

**Central Illustration:**
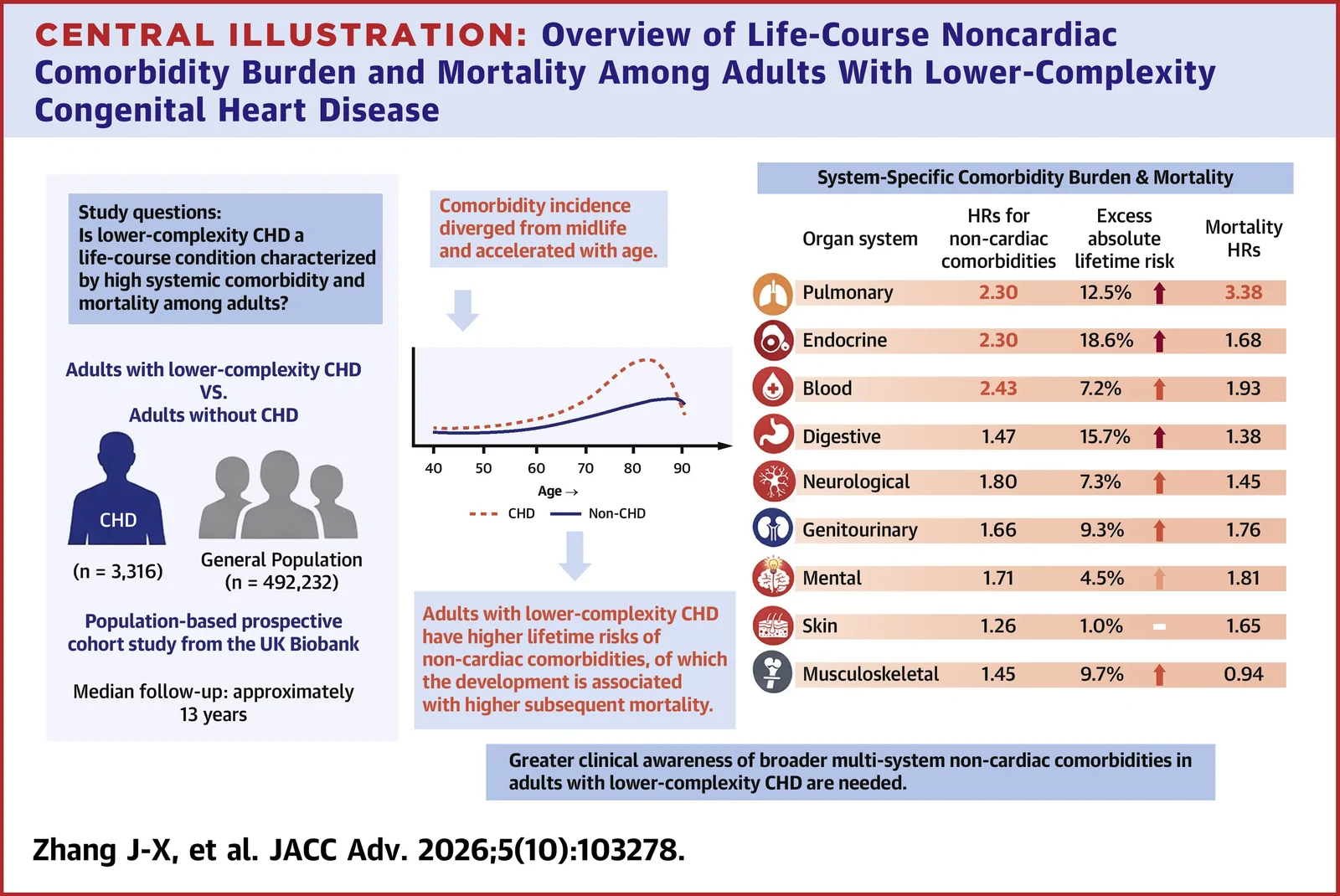
Overview of Life-Course Noncardiac Comorbidity Burden and Mortality Among Adults With Lower-Complexity Congenital Heart Disease Abbreviation as in Figure 1.

Several mechanisms may underlie our observations. First, genetic or in utero factors contributing to cardiac malformations may also impair the development of other organ systems, predisposing individuals to multisystem disease later in life.^21^ This has been supported by evidence linking increased risk of neurodevelopmental disorders with CHD.^22^ Meanwhile, maternal conditions like diabetes and obesity increase CHD risk and could confer lifelong metabolic vulnerabilities in offspring.^23^ Second, neurohormonal and autonomic activation, along with chronic hemodynamic alterations, may contribute to renal vulnerability in adults with CHD, even when asymptomatic.^24^^,^^25^ Third, persistent abnormalities in pulmonary function have been reported following repair of atrial septal defect and ventricular septal defect, potentially related to timing of surgery and the severity of CHD prior to surgery.^26^, ^27^, ^28^ Moreover, chronic left-to-right shunt and increased pulmonary flow may promote vascular remodeling and pulmonary arterial hypertension,^29^ representing a critical interface between CHD and pulmonary vascular pathology. Finally, unemployment or not studying, functional impairment, lower social support, and lower quality of life have been reported to be associated with depression and/or anxiety in adults with CHD, including patients with simple lesions.^30^ However, given the multifactorial nature of psychological distress across the lifespan, clinically meaningful anxiety and depression may not always be readily identifiable.^31^ Besides psychological distress, socioeconomic disadvantage has also been found associated with neurodevelopmental impairments in individuals with CHD.^32^ Notably, systemic vulnerability may extend beyond lesion complexity itself. Previous studies showed that patients with mild CHD were more often prefrail than those with complex lesions, suggesting that individuals with conditions traditionally considered benign may still be associated with functional vulnerability.^33^

As substantial advances have been made in CHD care since the 1950s, especially for transcatheter closure and cardiac surgery,^34^ contemporary patients with CHD may undergo complete repair earlier in life and therefore experience a shorter cumulative duration of unrepaired CHD than individuals represented in our cohort. Consequently, future longitudinal studies incorporating contemporary birth cohorts will be important to determine how advances in CHD diagnosis and treatment influence long-term noncardiac health outcomes. Until such evidence becomes available, our study provides direct evidence for contemporary adult survivors with lower-complexity CHD, for whom access to specialized adult CHD services remains limited even in regions with adequate interventional care.^35^ In addition, specialized CHD centers are uncommon in low- and middle-income countries, where Eisenmenger syndrome is still encountered, indicating that timely diagnosis and repair of CHD have not yet been achieved universally.^36^ Consequently, evidence from long-term follow-up cohorts such as ours may provide valuable insights for the growing population of adults with CHD in these regions.

## Strengths and limitations

Several strengths existed, such as the comprehensive assessment of nearly all major noncardiac organ systems, and application of both life-table methods and Cox proportional hazards models. Because the UKB cohort consists of individuals born between 1931 and 1971 and with long-term follow-up,^12^ it provides an opportunity to investigate long-term health trajectories among adults with lower-complexity CHD, a population that has historically received less attention in longitudinal outcome studies.

Several limitations of our study should also be acknowledged. First, participants in the UKB cohort tend to be healthier and more affluent than the general population,^37^ and were born during an era when neonatal and early-childhood interventions for CHD were less widely available, potentially limiting external generalizability. Second, because only participants aged 37 to 73 years were enrolled in the UKB, lifetime risk estimates from age 40 onward likely underestimate the true burden of noncardiac comorbidities across the full life course in individuals with lower-complexity CHD, particularly for outcomes that may begin earlier in adulthood, such as neurologic disease.^19^ Third, women were relatively under-represented in the lower-complexity CHD cohort of our study. Given previously reported sex differences in long-term survival in adults with CHD,^38^^,^^39^ the mortality findings may not be fully generalizable to women. Fourth, since genetic syndromes may co-occur with CHD and influence extracardiac comorbidity burden,^40^ their exclusion may reduce potential confounding but also limit generalizability to the broader CHD population. Finally, residual confounding cannot be fully excluded because of the observational design, despite multivariable adjustment and E-value analyses.

## Conclusions

This study provides quantitative, population-based evidence supporting that lower-complexity CHD, across both simple and moderate complexity lesions, is associated with a higher burden of multisystem noncardiac comorbidities accumulating progressively from midlife. The onset of comorbidities represents an association with higher subsequent mortality. These findings support greater clinical awareness of broader multisystem noncardiac comorbidities in adults with lower-complexity CHD.

Perspectives **COMPETENCY IN MEDICAL KNOWLEDGE:** Adults with lower-complexity CHD, despite being traditionally considered mild, experience a significantly increased burden of multisystem noncardiac comorbidities compared to those without CHD. Comorbidity incidence diverged from midlife and accelerated with age, which was associated with higher subsequent mortality.

**TRANSLATIONAL OUTLOOK:** Assessment of noncardiac comorbidities, particularly blood, endocrine, digestive, and pulmonary diseases, may facilitate more comprehensive lifelong care strategies in adults with lower-complexity CHD. Future studies are needed to validate these findings, clarify underlying mechanisms, and determine whether integrated preventive approaches can improve long-term outcomes.

## Funding support and author disclosures

This work was supported by 10.13039/501100001809National Natural Science Foundation of China (82500073, 82241020, 82300082); Noncommunicable Chronic Diseases-National Science and Technology Major Project (2024ZD0526700); and Shenzhen Medical Research Fund (C2404001). The funder had no role in study design, data collection, analysis, interpretation, or writing. The authors have reported that they have no relationships relevant to the contents of this paper to disclose.
