# Supplementary material for: Life-Course Noncardiac Comorbidity Burden and Mortality in Lower-Complexity Congenital Heart Disease
Source: JACC Adv. 2026 Sep 24;5(10):103278. doi: 10.1016/j.jacadv.2026.103278 (PMC13634985; doi:10.1016/j.jacadv.2026.103278)

SUPPLEMENTARY MATERIAL

Life-course Non-cardiac Comorbidity Burden and Mortality in Lower-Complexity Congenital Heart Disease

Corresponding author:
Zhi-Cheng Jing, MD, PhD
Professor, Department of Cardiology Guangdong Cardiovascular Institute, Guangdong Provincial People's Hospital,
Guangdong Academy of Medical Sciences, Southern Medical University. No. 106 Zhongshan 2nd Road, Guangzhou, 510080,
China.
Email: jingzhicheng@vip.163.com; jingzhicheng@gdph.org.cn

TABLE OF CONTENTS

SUPPLEMENTARY MATERIAL……………………………………………………………..1
TABLE OF CONTENTS……………………..…………………………………………1
STEERING COMMITTEE……………………………………………………………2
SUPPLEMENTARY TABLES………………………..……………………………………3
Table S1. Codes for Inclusion Criteria of Specific CHD Diagnosis and Non-specific but Possible CHD Diagnosis…………………………………………………………………………………..………3
Table S2. Codes for Exclusion Criteria…………………………………………………………………9
Table S3. Codes for Reclassifying Unconfirmed CHD as Non-CHD……………………..………………10
Table S4. Codes for Exclusion Criteria of Non-CHD…………………………..……………................12
Table S5. CHD Subgroup Definitions…………………………………….…………………………13
Table S6. Median Follow-up Time and Number of Individuals Across Systems…….……...……………14
Table S7. Codes for Diagnosis of Outcomes across Systems. ……………………………………………15
Table S8 E-values for the Associations between Lower-complexity CHD and First-time Non-cardiac Comorbidities by System……………………………………………………………………...….17
Table S9 Fine–Gray Subdistribution Hazard Models for First-time Non-cardiac Comorbidities by System.18
Table S10 Distribution of Identifiable Cardiac Intervention Records Across Lower-complexity CHD Subgroups…………………………………………………………………………………….……..19
Table S11 Subgroup-stratified Cox Analyses for Assessing Potential Influence of Intervention in Lower-complexity CHD……………………………………………………………………………………20
Table S12 Number of CHD Participants and All-cause Mortality Events Across System-specific Analyses……………………………………………………………………………………….…22
Figure S1. Cumulative Incidence of Non-cardiac Comorbidities across Systems …………………..........23
Figure S2. Age-specific Incidence Rates (per 100,000 person-years) of First-time Non-cardiac Comorbidity Occurrence for Each System Stratified by CHD Status……………………………………………………24
Figure S3. Cumulative Survival Rates Among Lower-complexity CHD Patients with or without System-specific Non-cardiac Comorbidities……..………………………………………………………..………25
Figure S4 Adjusted HRs and 95% CIs for All-cause Mortality associated with Incident Non-cardiac Comorbidities among Individuals with Lower-complexity CHD……………………………………….26

STEERING COMMITTEE

There is no Steering Committee in this study; all author names are mentioned in the manuscript: "Life-course Non-cardiac Comorbidity Burden and Mortality in Lower-Complexity Congenital Heart Disease"

SUPPLEMENTARY TABLES

Supplementary Tables S1–4. All Code Lists for Lower-complexity CHD

Supplementary Table S1 Codes for Inclusion Criteria of Specific CHD Diagnosis and Non-specific but Possible CHD Diagnosis

**Codes for Specific CHD Diagnosis**						
**Diagnosis**	**ICD 9**	**ICD 10**	**OPCS 4**	**Self-reported Medical History**	**Self-reported Surgical History**	**Diagnostic Age (in years) for Inclusion**
Truncus arteriosus	745.0*	Q20.0	L01*, K29.6, K29.7			
Transposition of great vessels	745.1*	Q20.3	K05.1, K05.2, K05.8, K05.9, K06.1, K06.2, K06.3, K06.4, K06.8, K06.9, K18.6			
Tetralogy of Fallot	745.2*	Q21.3	K04.1, K04.2, K04.3, K04.4, K04.5, K04.6, K04.8, K04.9, K24.2, K18.3, K19.3			
Ventricular septal defect	745.4*	Q21.0	K11.1, K11.2, K11.3, K11.4, K11.5, K11.6, K11.7, K11.8, K11.9, K13. 1, K13.2			
Atrial septal defect/Patent foramen ovale	745.5*	Q21.1	K09.4, K10*, K13.3, K13.4, K14.3, K16.3			
Atrioventricular septal defect	745.62, 745.63	Q21.2	K09.1, K09.2, K09.3, K09.4, K09.5, K09.6, K09.8, K09.9			

Aortopulmonary septum defect		Q21.4	L01.4			
Other congenital malformations of cardiac septum	745.60, 745.61, 745.68, 745.69, 745.8, 745.9	Q21.8, Q21.9	K12.1, K12.2, K12.3, K12.4, K12.5, K12.8, K12.9, K13.5, K13.8, K13.9, K14.8, K14.9, K15.8, K15.9, K16.8, K16.9			
Patent ductus arteriosus	747.0	Q25.0	L02.1, L02.2, L02.3, L02.4, L02.8, L02.9, L03.1			
Congenital tricuspid stenosis or atresia	746.1	Q22.4, Q22.6				
Other congenital malformations of tricuspid valve		Q22.8, Q22.9				
Ebstein's anomaly	746.2	Q22.5				
Aortic coarctation	747.1*	Q25.1				
Aortic atresia	747.20, 747.21	Q25.2, Q25.3	L23.7			
Congenital aneurysm of aorta	747.27					
Unspecified congenital anomaly of aorta	747.23, 747.24, 747.25, 747.26, 747.28, 747.29	Q25.4	K37.4			
Congenital subaortic stenosis		Q24.4				
Congenital aortic stenosis	746.3	Q23.0				
Congenital aortic insufficiency	746.4	Q23.1				
Congenital mitral stenosis	746.5	Q23.2				

Congenital mitral insufficiency	746.6	Q23.3				
Other congenital malformations of aortic or mitral valves		Q23.8, Q23.9				
Hypoplastic Left Heart Syndrome (HLHS)	746.7	Q23.4	K17.5			
Congenital pulmonary stenosis or atresia	746.01, 746.83, 747.32, 746.00, 747.30, 747.31	Q22. 1, Q25.6, Q24.3, Q22.0, Q25.5				
Congenital pulmonary valve insufficiency	746.02	Q22.2				
Other malformations of pulmonary valve or artery	746.08, 746.09, 747.33, 747.34, 747.38, 747.39	Q25.71, Q25.72, Q22.3, Q25.7, Q25.79	L10.1, L10.2, L10.3, L10.4, L10.8, L10.9, L12. 1, L12.2, L12.3			
Congenital coronary vessel anomalies		Q24.5				
Congenital heart block	746.87	Q24.6	K57.7			
Total anomalous pulmonary venous return	747.42	Q26.2	K07.1, K07.2, K07.3, K07.8, K07.9			
Partial anomalous pulmonary venous return	747.43	Q26.3, Q26.4	K20.2			
Cor biloculare	745.7					
Cor triatriatum	746.82	Q24.2	K20.3			
Double outlet right ventricle		Q20.1	K08.1, K08.2, K08.3			

Double outlet left ventricle		Q20.2	K08.4			
Double inlet ventricle		Q20.4				
Discordant atrioventricular connection		Q20.5				
Isomerism of atria	746.80, 746.81, 759.3, 759.30, 759.31, 759.32	Q20.6, Q24.0, Q24.1, Q89.3				
Unspecified congenital malformations of cardiac chambers		Q20.8, Q20.9	K08.8, K08.9			
Unspecified acyanotic congenital heart disease	746.88, 746.90, 746.91, 746.92, 746.99, 747.40, 747.41	Q24.8, Q24.9, Q26.0, Q26.1	K18.1, K18.5, K18.7, K18.8, K18.9, K19.1, K19.6, K19.8, K19.9			
Unspecified cyanotic defect	746.93, 745.3	Q25.8, Q25.9	K19.2, K18.2, K24.1, K14.1, K14.2, K14.4, K14.5, K15.1, K15.2, K16.1, K16.2, K16.3, K16.4, L05.1, L05.2, L05.3, L05.4, L05.8, L05.9, L07.1, L07.2, L07.3, L07.4, L07.5, L07.8, L07.9, L08.1, L08.2, L08.3, L08.4, L08.6, L08.7, L08.8, L08.9, L09.1, L09.2, L03.2, L09.8, L09.9, K17.1, K17.2, K17.6, K17.7, K17.8,			

			K17.9, K17.3, K17.4, L06.1, L06.2, L06.3, L06.4, L06.5, L06.6, L06.7, L06.8, L06.9			
**Codes for non-Specific CHD with Diagnostic Ages Indicative of Congenital Etiology**						
**Diagnosis**	**ICD 9**	**ICD 10**	**OPCS 4**	**Self-reported Medical History**	**Self-reported Surgical History**	**Diagnostic age (in years) for inclusion**
Aortic stenosis		I35.0	K31.2, K32.2, K35.2	1490		<60
Aortic insufficiency		I35.1, I35.2		1587		<60
Subaortic stenosis			K37.3			
Aortic valve replacement			K26.1, K26.2, K26.3, K26.4, K26.5, K26.8, K26.9, K30.2		1099	<65
Unspecified aortic valve condition	424.1			1586		
Pulmonary insufficiency		I37.1, I37.2				

Pulmonary stenosis		I37.0				
Pulmonary valve defect or repair	424.3		K28.1, K28.2, K28.3, K28.4, K28.5, K28.8, K28.9, K30.4, K35.7, K31.4, K32.4, K35.4, K35.6			
Unspecified heart surgery					1069	<18

*Inclusive of diagnostic codes with trailing digits.

Abbreviations: CHD = congenital heart disease; ICD 9 = International Classification of Diseases, 9th revision; ICD 10 = International Classification of Diseases, 10th revision; OPCS 4 = Office of Population Censuses and Surveys Classification of Interventions and Procedures version 4

Supplementary Table S2 Codes for Exclusion Criteria

Diagnosis	ICD 9	ICD 10	OPCS 4	Self-reported Medical History	Self-reported Surgical History
Genetic syndromes	758*, 759.5	Q85. 1, Q90*, Q91*, Q92*, Q93*, Q96*, Q87.5, Q87.8, Q87.81, Q87.89			
Connective tissue disorders (including Marfan's disease)	756.85	Q79.6, Q87.4, Q87.40, Q87.410, Q87.418, Q87.42, Q87.43		1373	
Traumatic injury of heart			K55.6		

*Inclusive of diagnostic codes with trailing digits.

Abbreviations: ICD 9 = International Classification of Diseases, 9th revision; ICD 10 = International Classification of Diseases, 10th revision; OPCS 4 = Office of Population Censuses and Surveys Classification of Interventions and Procedures version 4

Supplementary Table S3 Codes for Reclassifying Unconfirmed CHD as Non-CHD

**Diagnosis**	**ICD 9**	**ICD 10**	**OPCS 4**	**Self-reported Medical History**	**Self-reported Surgical History**	**Criteria Applied for:**
Rheumatic heart disease	390, 390.9, 391*, 392*, 393*, 394. 1, 395.0, 395. 1, 395.2, 397. 1, 397.9, 398, 398.0, 398.9	I01*, I05*, I06*, I07*, I08*, I09*		1479		Unconfirmed pulmonary valve defects, unconfirmed aortic valve defects, heart surgery prior to age 18
Mitral valve disease	394.0, 394.2, 394.9	I34.2	K31. 1, K32. 1, K35. 1, K25. 1, K25.2, K25.3, K25.4, K25.5, K25.8, K25.9, K30.1	1584, 1585, 1489	1100	Unconfirmed pulmonary valve defects, unconfirmed aortic valve defects, heart surgery prior to age 18
Endocarditis	093.2, 421, 421.0, 421. 1, 421.9, 424.9	I33, I33.0, I33.9, I38, I39, I39.0, I39. 1, I39.3, I39.4, I39.8, A32.82, A39.51, A52.03, B33.21, B37.6, M32.11	K34.4			Unconfirmed pulmonary valve defects, unconfirmed aortic valve defects, heart surgery prior to age 18
Lupus with organ involvement		M32.1				Unconfirmed pulmonary valve defects, unconfirmed aortic valve defects, heart surgery prior to age 18

Malignant carcinoid syndrome		C7A.0*, C7B.0*		1432		Unconfirmed pulmonary valve defects, unconfirmed aortic valve defects, heart surgery prior to age 18
Aortitis	093. 1, 093.0, 447.7, 446.7, 446.5	I79.0, I79. 1, M31.4, M31.5, M31.6, A52.02				Unconfirmed aortic valve defects, heart surgery prior to age 18
Pulmonary hypertension	416.0	I27.0, I27.2				Unconfirmed pulmonary valve defects, heart surgery prior to age 18
Post-Myocardial Infarction Septal Defect		I23. 1, I23.2				Atrial septal defects

*Inclusive of diagnostic codes with trailing digits.

Abbreviations: CHD = congenital heart disease; ICD 9 = International Classification of Diseases, 9th revision; ICD 10 = International Classification of Diseases, 10th revision; OPCS 4 = Office of Population Censuses and Surveys Classification of Interventions and Procedures version 4

Supplementary Table S4 Codes for Exclusion Criteria of Non-CHD

**Diagnosis**	**ICD 9**	**ICD 10**	**OPCS**	**Self-reported Medical History**	**Self-reported Surgical History**
Endocarditis	093.2, 421, 421.0, 421. 1, 421.9, 424.9	I33, I33.0, I33.9, I38, I39, I39.0, I39. 1, I39.3, I39.4, I39.8, A32.82, A39.51, A52.03, B33.21, B37.6, M32.11	K34.4		
Thoracic aortic dissection or aneurysm	441. 1, 441.2	I71. 1, I71.2, I71.01	L27.3, L28.3		
Aortic root dilation			K33, K33. 1, K33.2, K33.3, K33.4, K33.5, K33.6, K33.8, K33.9		
Pulmonary Hypertension	416	I27.0, I27.2			
Heart transplant	V42. 1, V43.2	Z94.1	K01. 1, K01.2, K01.8, K01.9, K02. 1, K02.2, K02.3, K02.4, K02.5, K02.6, K02.8, K02.9		1098
Hypertrophic cardiomyopathy	425.1	I42. 1, I42.2	K24.5, K24.6, K24.7	1588	

Abbreviations: CHD = congenital heart disease; ICD 9 = International Classification of Diseases, 9th revision; ICD 10 = International Classification of Diseases, 10th revision; OPCS 4 = Office of Population Censuses and Surveys Classification of Interventions and Procedures version 4

Supplementary Table S5 CHD Subgroup Definitions

	No. (%)
**Complex CHD**	**43 (100)**
Double-outlet ventricle, Interrupted aortic arch, Single ventricle, TGA, Truncus arteriosus	18 (41.9)
Unspecified cyanotic lesion	25 (58.1)
**Lower-complexity CHD**	**3316 (100)**
**Simple CHD**	**1055 (31.8)**
Left-to-right shunt (ASD, VSD, PDA)	1036 (31.2)
Pulmonic stenosis	19 (0.6)
**Moderate complexity CHD**	**1821 (54.9)**
Left sided lesions (Congenital aortic valve disease, Congenital mitral valve disease, Coarctation of the aorta)	1421(42.9)
Right sided lesions (Ebstein anomaly, Peripheral pulmonary artery stenosis, Congenital pulmonary valve insufficiency)	240 (7.2)
Other moderate lesions (TOF, APVC partial or total, Coronary artery malformation, AVSD, Aortopulmonary septal defect)	160 (4.8)
**Other lower-complexity CHD**	**440 (13.3)**
Conduction defect, Dextrocardia, Levocardia	65 (2.0)
Unspecified CHD	375 (11.3)

Abbreviations: ASD = atrial septal defect; APVC = anomalous pulmonary venous connection; AVSD = atrioventricular septal defect; CHD = congenital heart disease; PDA = patent ductus arteriosus; TOF = tetralogy of Fallot; TGA = transposition of the great arteries; VSD = ventricular septal defect

Supplementary Table S6 Median Follow-up Time and Number of Individuals Across Systems

Systems	No.	No. of Events (%)	Follow-up Time (median [IQR])
**Neurological**	421364	59806 (14.19)	13.46 [12.51, 14.24]
**Pulmonary**	387003	70411 (18.19)	13.41 [12.46, 14.23]
**Endocrine**	369799	97921 (26.48)	13.25 [11.74, 14.17]
**Mental**	411232	59705 (14.52)	13.49 [12.51, 14.27]
**Digestive**	312383	125475 (40.17)	12.76 [7.27, 13.98]
**Genitourinary**	332612	86685 (26.06)	13.22 [11.12, 14.15]
**Blood**	469201	48693 (10.38)	13.54 [12.65, 14.27]
**Skin**	366384	71447 (19.50)	13.35 [12.36, 14.21]
**Musculoskeletal**	283476	98943 (34.90)	12.94 [7.56, 14.04]

Abbreviations: IQR = interquartile range

Supplementary Table S7 Codes for Diagnosis of Outcomes across Systems

Systems	ICD 10	Most Frequent ICD 10 Codes & Diagnoses		No. (%) of Events in CHD
**Neurological**	G00–G99	G47	Sleep disorders	105 (16.38)
		G45	Transient cerebral ischaemic attacks and related syndromes	72 (11.23)
		G56	Mononeuropathies of upper limb	64 (9.98)
		G43	Migraine	55 (8.58)
		G81	Hemiplegia	46 (7.18)
		G40	Epilepsy	36 (5.62)
**Pulmonary**	J00–J99 (except J00-06 & J30-39)	J22	Unspecified acute lower respiratory infection	195 (21.57)
		J90	Pleural effusion, not elsewhere classified	167 (18.47)
		J18	Pneumonia, organism unspecified	164 (18.14)
		J98	Other respiratory disorders	74 (8.19)
		J44	Other chronic obstructive pulmonary disease	70 (7.74)
		J45	Asthma	70 (7.74)
**Endocrine**	E01–E89	E78	Disorders of lipoprotein metabolism and other lipidaemias	461 (41.27)
		E66	Obesity	205 (18.35)
		E87	Other disorders of fluid, electrolyte and acid-base balance	121 (10.83)
		E11	Non-insulin-dependent diabetes mellitus	83 (7.43)
		E03	Other hypothyroidism	51 (4.57)
		E86	Volume depletion	39 (3.49)
**Mental and behavioural**	F00–F99	F17	Mental and behavioural disorders due to use of tobacco	167 (27.07)
		F32	Depressive episode	126 (20.42)
		F41	Other anxiety disorders	89 (14.42)
		F10	Mental and behavioural disorders due to use of alcohol	71 (11.51)
		F05	Delirium, not induced by alcohol and other psychoactive substances	53 (8.59)
		F03	Unspecified dementia	20 (3.24)
**Digestive**	K00–K93	K57	Diverticular disease of intestine	121 (11.82)

		K21	Gastro-oesophageal reflux disease	93 (9.08)
		K29	Gastritis and duodenitis	82 (8.01)
		K59	Other functional intestinal disorders	76 (7.42)
		K40	Inguinal hernia	73 (7.13)
		K62	Other diseases of anus and rectum	51 (4.98)
**Genitourinary**	N00–N99	N17	Acute renal failure	177 (19.78)
		N18	Chronic renal failure	148 (16.54)
		N39	Other disorders of urinary system	110 (12.29)
		N40	Hyperplasia of prostate	108 (12.07)
		N28	Other disorders of kidney and ureter, not elsewhere classified	32 (3.58)
		N32	Other disorders of bladder	30 (3.35)
**Blood**	D50–D89	D64	Other anaemias	320 (44.44)
		D50	Iron deficiency anaemia	200 (27.78)
		D69	Purpura and other haemorrhagic conditions	53 (7.36)
		D70	Agranulocytosis	30 (4.17)
		D68	Other coagulation defects	26 (3.61)
		D75	Other diseases of blood and blood-forming organs	16 (2.22)
**Skin and subcutaneous**	L00–L99	L03	Cellulitis	99 (16.67)
		L98	Other disorders of skin and subcutaneous tissue, not elsewhere classified	62 (10.44)
		L30	Other dermatitis	53 (8.92)
		L57	Skin changes due to chronic exposure to nonionising radiation	47 (7.91)
		L82	Seborrhoeic keratosis	46 (7.74)
		L89	Decubitus ulcer	34 (5.72)
**Musculoskeletal**	M00–M99	M19	Other arthrosis	95 (11.38)
		M79	Other soft tissue disorders, not elsewhere classified	86 (10.30)
		M54	Dorsalgia	74 (8.86)
		M17	Gonarthrosis [arthrosis of knee]	66 (7.90)
		M10	Gout	54 (6.47)
		M13	Other arthritis	51 (6.11)

Abbreviations: CHD = congenital heart disease; ICD 10 = International Classification of Diseases, 10th revision.

Supplementary Table S8 E-values for the Associations between Lower-complexity CHD and First-time Non-cardiac Comorbidities by System

Systems	HR (95% CI)	E-value	E-value for Lower 95% CI
**Neurological**	**1.80 (1.66, 1.96)**	3.01	2.71
**Pulmonary**	**2.30 (2.14, 2.46)**	4.02	3.70
**Endocrine**	**2.30 (2.16, 2.44)**	4.02	3.74
**Mental**	**1.71 (1.57, 1.86)**	2.81	2.51
**Digestive**	**1.47 (1.37, 1.56)**	2.29	2.09
**Genitourinary**	**1.66 (1.55, 1.78)**	2.71	2.47
**Blood**	**2.43 (2.24, 2.63)**	4.29	3.91
**Skin**	**1.26 (1.15, 1.37)**	1.82	1.57
**Musculoskeletal**	**1.45 (1.35, 1.55)**	2.25	2.03

Abbreviations: CHD = congenital heart disease; HR = hazard ratio; CI = confidence interval

Supplementary Table S9 Fine–Gray Subdistribution Hazard Models for First-time Non-cardiac Comorbidities by System

**Systems**	**HR (95% CI) in Cox proportional hazards models**	**SHR (95% CI) in Fine–Gray subdistribution hazard models**
**Neurological**	**1.80 (1.66, 1.96)**	1.77 (1.62, 1.92)
**Pulmonary**	**2.30 (2.14, 2.46)**	2.28 (2.13, 2.44)
**Endocrine**	**2.30 (2.16, 2.44)**	2.27 (2.13, 2.42)
**Mental**	**1.71 (1.57, 1.86)**	1.68 (1.54, 1.83)
**Digestive**	**1.47 (1.37, 1.56)**	1.46 (1.37, 1.55)
**Genitourinary**	**1.66 (1.55, 1.78)**	1.64 (1.54, 1.76)
**Blood**	**2.43 (2.24, 2.63)**	2.39 (2.20, 2.58)
**Skin**	**1.26 (1.15, 1.37)**	1.24 (1.14, 1.35)
**Musculoskeletal**	**1.45 (1.35, 1.55)**	1.42 (1.33, 1.53)

Abbreviations: CHD = congenital heart disease; HR = hazard ratio; SHR = subdistribution hazard ratio; CI = confidence interval

Supplementary Table S10 Distribution of Identifiable Intervention Records Across Lower-complexity CHD Subgroups

Lower-complexity CHD Subgroups	No. of Participants	No. of Participants with Identifiable Intervention Records (%)	Identifiable Intervention Code (OPCS4 codes & Self-reported data)
**Simple CHD**			
Left-to-right shunt	1036	319 (30.8)	K10*; K11*; K12.1; K12.4; K12.8; K13.1; K13.2; K13.3; K13.4; K16.8; L02*; L03.1
Pulmonic stenosis	19	1 (5.3)	1069
**Moderate complexity CHD**			
Left sided lesions	1421	406 (28.6)	K26*; K30.2; K37.3; 1069; 1099
Right sided lesions	240	46 (19.2)	K10.4; K28*; K35.4; L10*; 1069
Other moderate lesions	160	46 (28.8)	K04*; K07*; K09*; K10.1; K10.2; K10.4; K10.8; K10.9; K11.1; K11.4; K13.3; K18.3; K20.2
**Other lower-complexity CHD**			
Conduction defect, Dextrocardia, Levocardia	65	8 (12.3)	K57.7
Unspecified CHD	375	185 (49.3)	K12.1; K12.2; K12.5; K14.8; K16.8; 1069

*Inclusive of codes with trailing digits

Codes represent identifiable intervention records observed among participants within each subgroup and include both surgical and catheter-based interventions. Because some participants had multiple congenital cardiac defects, intervention codes are not necessarily specific to the lesion defining subgroup assignment.

Abbreviations: CHD = congenital heart disease

Supplementary Table S11 Subgroup-stratified Cox Analyses for Assessing Potential Influence of Intervention in Lower-complexity CHD

Systems	Comparison	No. of Events/No. of Participants	HR (95% CI)	P Value
**Neurological disease**	**Non-CHD (Control)**	**59165/418803**	**1**	**ref**
	**Lower-complexity CHD**	**641/2561**	**1.80 (1.66, 1.96)**	**<0.001**
	without intervention records	472/1778	1.91 (1.73, 2.10)	<0.001
	with intervention records	169/783	1.55 (1.32, 1.83)	<0.001
**Pulmonary disease**	**Non-CHD (Control)**	**69507/384640**	**1**	**ref**
	**Lower-complexity CHD**	**904/2363**	**2.30 (2.14, 2.46)**	**<0.001**
	without intervention records	673/1634	2.45 (2.26, 2.65)	<0.001
	with intervention records	231/729	1.93 (1.68, 2.22)	<0.001
**Endocrine disease**	**Non-CHD (Control)**	**96804/367632**	**1**	**ref**
	**Lower-complexity CHD**	**1117/2167**	**2.30 (2.16, 2.44)**	**<0.001**
	without intervention records	853/1533	2.53 (2.35, 2.71)	<0.001
	with intervention records	264/634	1.75 (1.54, 1.99)	<0.001
**Mental and behavioural disease**	**Non-CHD (Control)**	**59088/408557**	**1**	**ref**
	**Lower-complexity CHD**	**617/2675**	**1.71 (1.57, 1.86)**	**<0.001**
	without intervention records	463/1847	1.89 (1.71, 2.08)	<0.001
	with intervention records	154/828	1.31 (1.10, 1.56)	0.002
**Digestive disease**	**Non-CHD (Control)**	**124451/310479**	**1**	**ref**
	**Lower-complexity CHD**	**1024/1904**	**1.47 (1.37, 1.56)**	**<0.001**
	without intervention records	746/1317	1.56 (1.45, 1.68)	<0.001
	with intervention records	278/587	1.25 (1.10, 1.42)	<0.001
**Genitourinary disease**	**Non-CHD (Control)**	**85790/330414**	**1**	**ref**
	**Lower-complexity CHD**	**895/2198**	**1.66 (1.55, 1.78)**	**<0.001**
	without intervention records	653/1525	1.73 (1.59, 1.87)	<0.001
	with intervention records	242/673	1.51 (1.32, 1.72)	<0.001
**Blood disease**	**Non-CHD (Control)**	**47973/466187**	**1**	**ref**
	**Lower-complexity CHD**	**720/3014**	**2.43 (2.24, 2.63)**	**<0.001**
	without intervention records	533/2114	2.52 (2.30, 2.76)	<0.001
	with intervention records	187/900	2.20 (1.88, 2.57)	<0.001
**Skin and subcutaneous disease**	**Non-CHD (Control)**	**70853/363962**	**1**	**ref**
	**Lower-complexity CHD**	**594/2422**	**1.26 (1.15, 1.37)**	**<0.001**
	without intervention records	432/1678	1.30 (1.18, 1.44)	<0.001

	with intervention records	162/744	1.15 (0.97, 1.35)	0.10
**Musculoskeletal disease**	**Non-CHD (Control)**	**98108/281682**	**1**	**ref**
	**Lower-complexity CHD**	**835/1794**	**1.45 (1.35, 1.55)**	**<0.001**
	without intervention records	594/1215	1.54 (1.41, 1.67)	<0.001
	with intervention records	241/579	1.26 (1.10, 1.43)	<0.001

Abbreviations: CHD = congenital heart disease; HR = hazard ratio; CI = confidence interval

Supplementary Table S12 Number of CHD Participants and All-cause Mortality Events Across System-specific Analyses

Systems	No. of CHD Participants	No. of All-cause Mortality Events
**Neurological**	2561	483
**Pulmonary**	2363	398
**Endocrine**	2167	317
**Mental**	2675	501
**Digestive**	1904	317
**Genitourinary**	2198	417
**Blood**	3014	571
**Skin**	2422	469
**Musculoskeletal**	1794	321

Abbreviations: CHD = congenital heart disease

Supplementary Figure S1 Cumulative Incidence of Non-cardiac Comorbidities across Systems

Abbreviations: CHD = congenital heart disease.

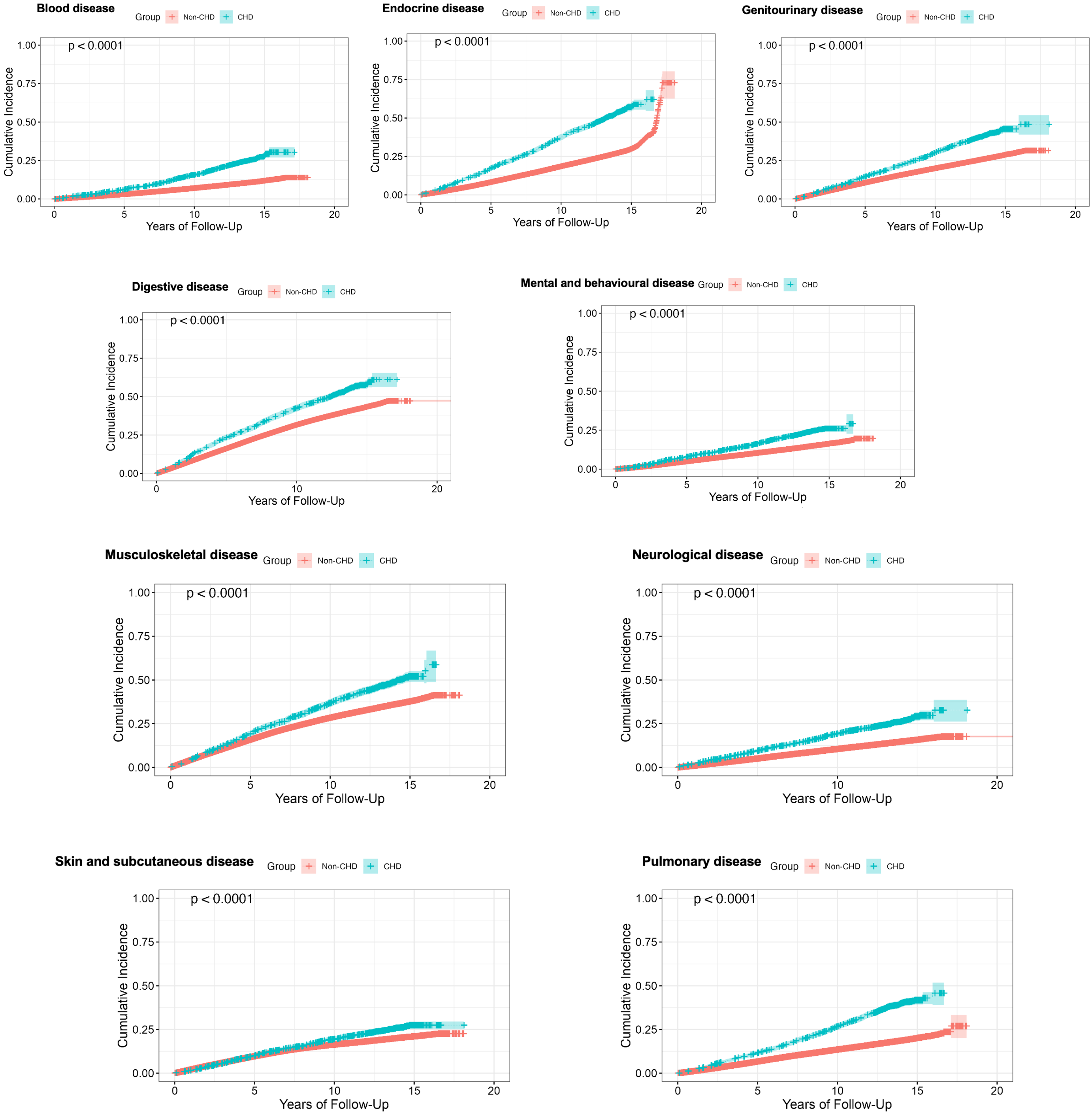

Supplementary Figure S2 Age-specific Incidence Rates (per 100,000 person-years) of First-time Non-cardiac Comorbidity Occurrence for Each System Stratified by CHD Status.

Abbreviations: CHD = congenital heart disease.

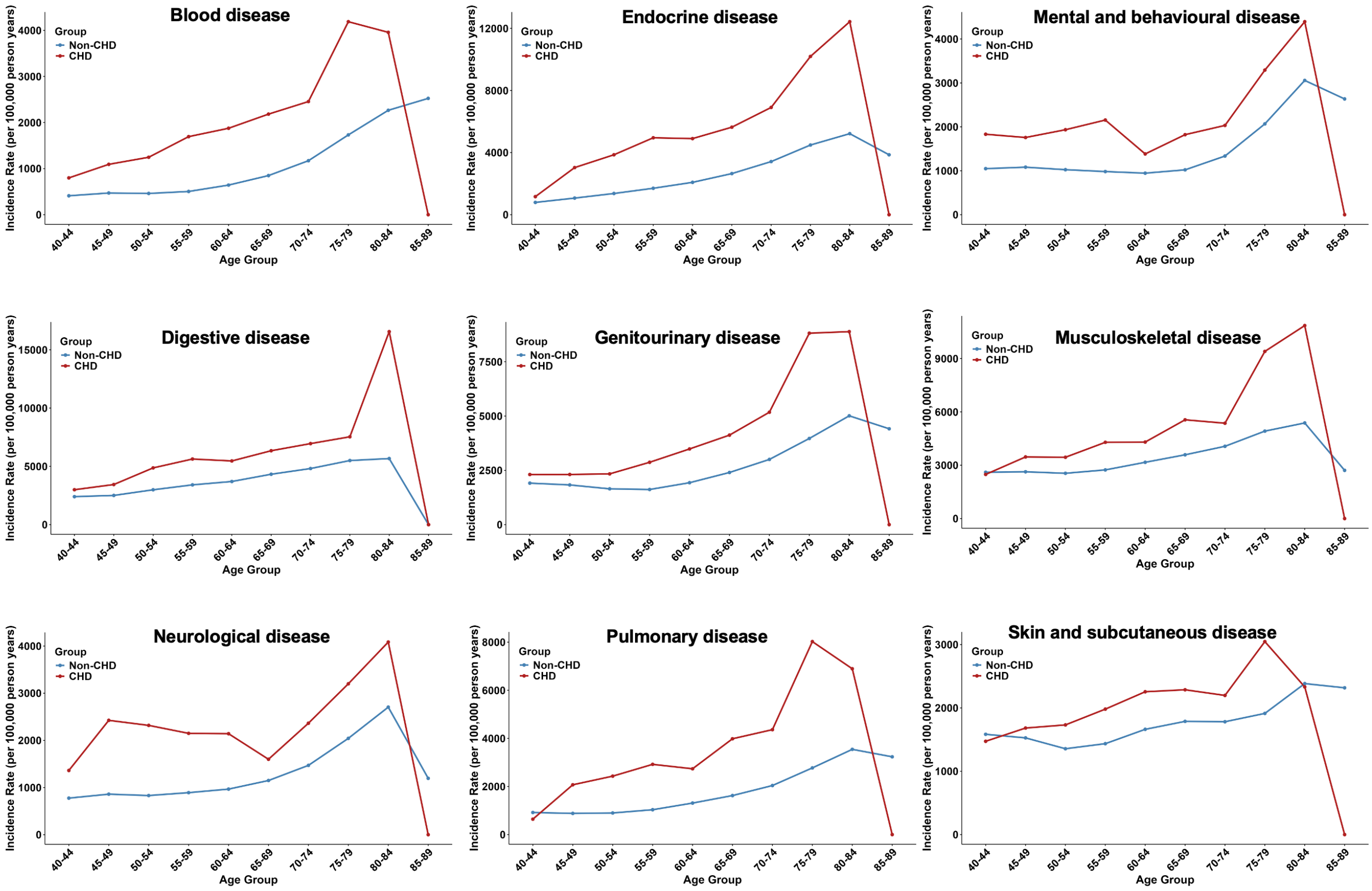

Supplementary Figure S3 Cumulative Survival Rates Among Lower-complexity CHD Patients with or without System-specific Non-cardiac Comorbidities

Abbreviations: CHD = congenital heart disease.

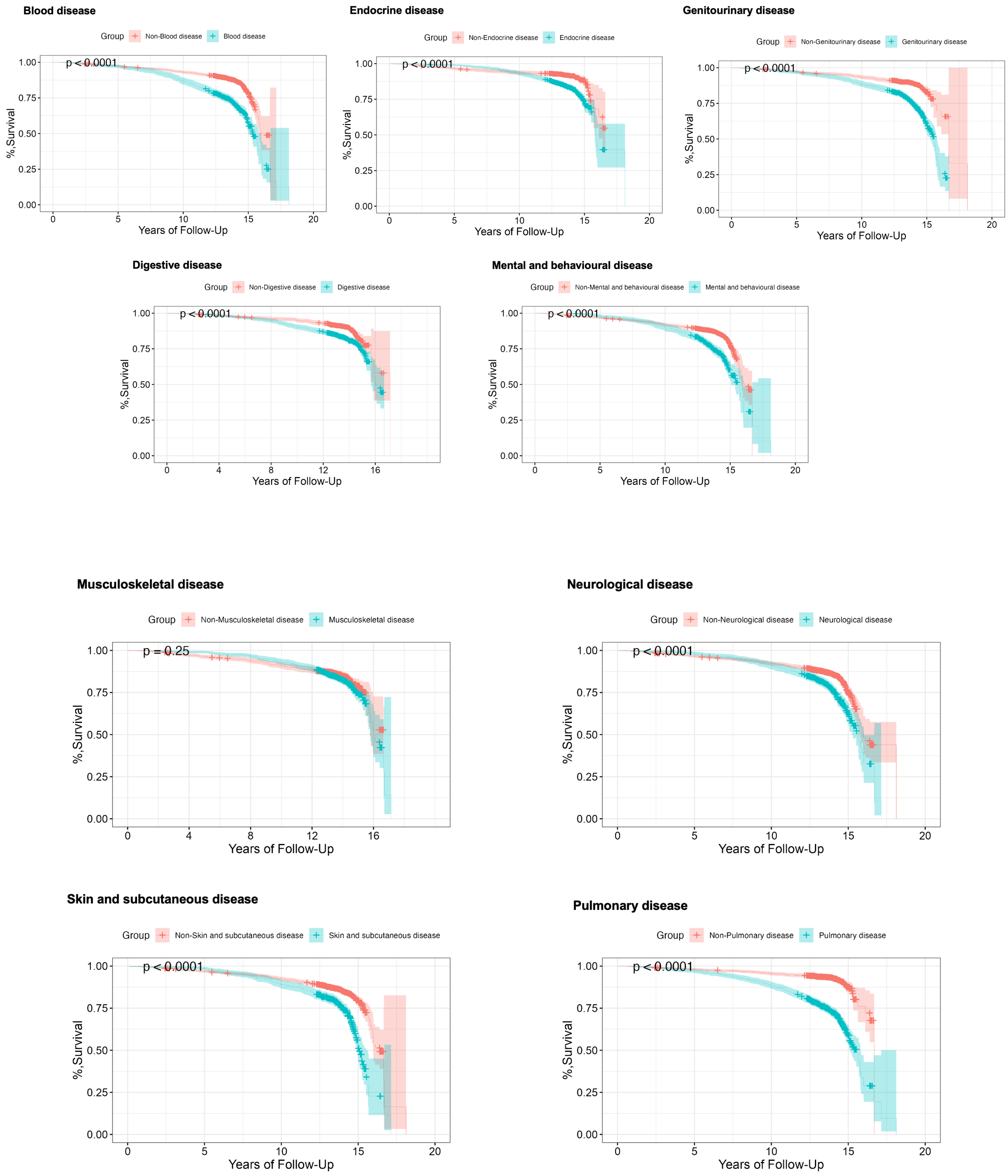

Supplementary Figure S4 Adjusted HRs and 95% CIs for All-cause Mortality associated with Incident Non-cardiac Comorbidities among Individuals with Lower-complexity CHD

Models were estimated using time-varying Cox proportional hazards models and adjusted for age, sex, ethnicity, Townsend deprivation index, body mass index, smoking status, alcohol consumption, and physical activity. All comparisons were statistically significant ($p < 0.05$). Abbreviations: HR = hazard ratio; CI = confidence interval; CHD = congenital heart disease.

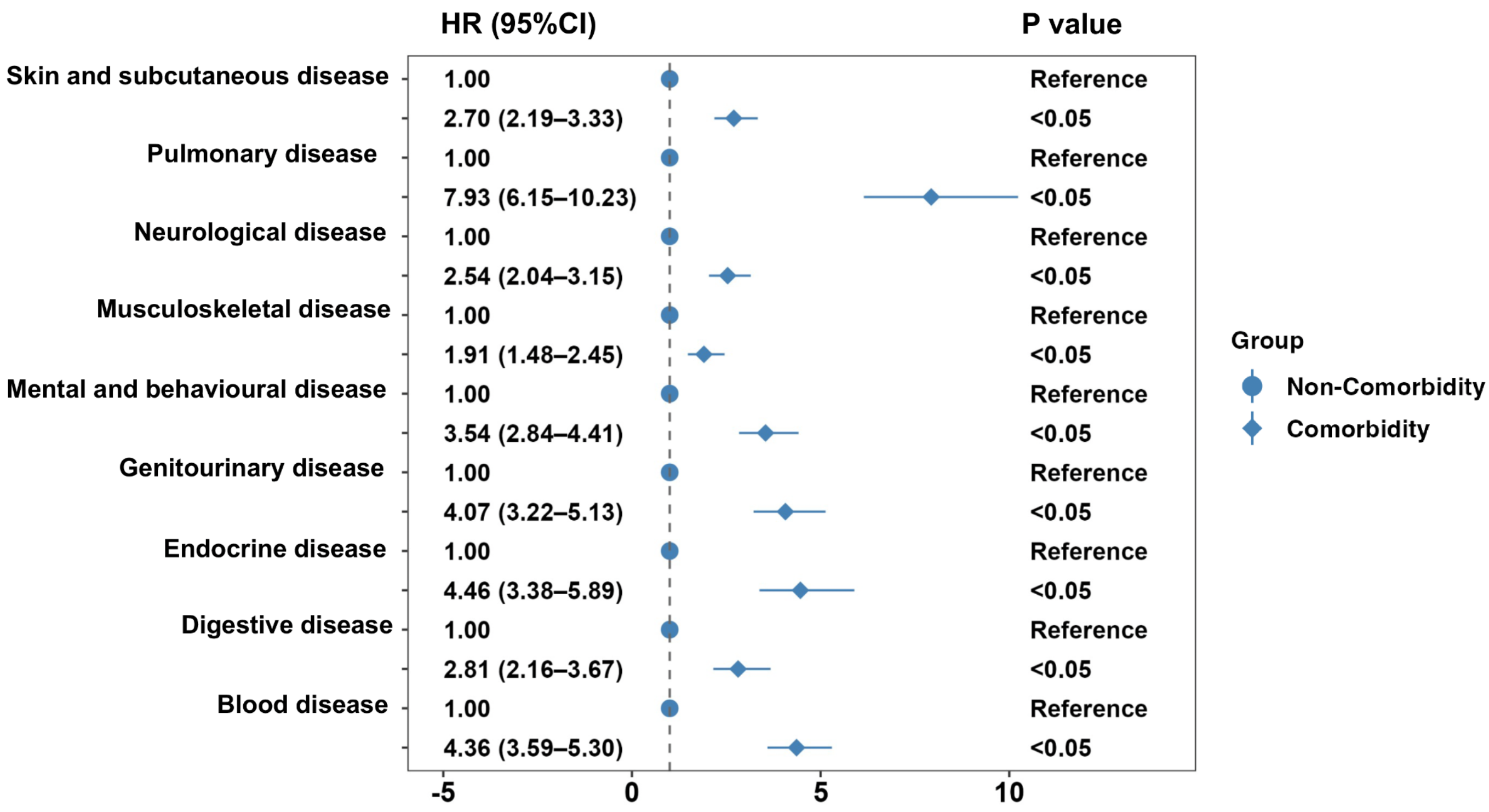

Supplement: Supplementary file 1 [file mmc1.pdf]
